# Monthly Summary

**Published:** 1894-02

**Authors:** 


### Monthly Summary.

A SPLENDID OPERATION.—The surgeons at the Roosevelt Hospital, New York, have just finished a marvel of dental surgery. Last August Mrs. S. G. Bedwell, of Yazoo City, Miss., came here with her sixteen-year-old daughter, who had a much-deformed face. When the young lady was four and a half years old she lost almost the entire lower jaw on the right side by necrosis, the bone coming away almost entirely by the sloughing away of the tissues in the neck under the angle of the jaw, and very little through surgical interference. The periostium was thus left intact and had finally filled up with a new growth of bone, which, however, did not grow any longer than a four-and-a-half-year-old child's jaw should be. The development of the jaw on the other side of the face being normal, it had pushed her chin around so much that it was under her right cheek, instead of the centre of her face.

The examining surgeon determined that if the new growth of bone was sawed through it would be possible to force the chin around to the front of the face, and hold it there permanently. The operation, was performed by Dr. Frank Hartley, at Roosevelt Hospital. He made an incision in the neck under the angle of the jaw, sawing the bone squarely in two. Gold and rubber plates and appliances were then inserted and the girl left for home to-day with her face once more in a normal position, its features regular and attractive. —*Baltimore Sun.*

---

DENTAL DOTS DISTILLED.—By D. V. Beacock, Brockville, Ont.—Ethylate of sodium is good to use on hypertrophied gums, free from pain and danger even if used in excess.

Cocaine dissolved in chloroform, one grain to one-eighth ounce of chloroform, is good to extirpate pulps without pain.

Take a small-sized sewing needle; at the distance of, say, three-quarters of an inch from the point, bend into the form of an S, the point of the needle forming the long leg; useful in filling labial cavities under the gum; stick the point into the neck of the tooth below the rubber dam, just above the edge of the cavity, lift the upper edge of dam over the eye end of the needle, and the resiliency of the rubber will keep the needle in place and the cavity dry. It is far ahead of any clamp for the above purpose. To prevent the eye of the needle penetrating the dam, put a little bead of shellac on the end.—*Texas Dental Journal.*

---

REMARKABLE TREPANNING.—Major John L. Hays, upon whom an operation was performed at the Allegheny general hospital, is now said to be out of danger, and his complete recovery is expected. Last October Major Hays was knocked senseless by a blow over the head with a chair. He became a violent maniac, and he was seized with as many as 100 convulsions in a day. Trepanning of the skull was decided upon. Upon removal of a portion of the skull a sack of pus was found in the membrane of the brain, the pressing down of which had caused all the trouble.—*Daily Ledger.*

---

A GENTLEMAN who was in Madrid for a number of years—Dr. Thomas—devised a plan for destroying pulps that seems so admirable that I want to tell it to you. He puts his arsenic, morphine and cinnamon together, and, having chopped up finely a quantity of cotton, mixes the medicament with it, and fills a bottle with the combination. It is ready for use whenever required, and is very comforting and quieting if the pulp is in a state of irritation. This preparation will not ooze out on the gum. I have been using it for five or six years.—*E. A. Bogue, Inter.*

